# Determining the serotype composition of mixed samples of pneumococcus using whole-genome sequencing

**DOI:** 10.1099/mgen.0.000494

**Published:** 2020-12-23

**Authors:** James R. Knight, Eileen M. Dunne, E. Kim Mulholland, Sudipta Saha, Catherine Satzke, Adrienn Tothpal, Daniel M. Weinberger

**Affiliations:** ^1^​ Department of Genetics, Yale School of Medicine, New Haven, Connecticut, USA; ^2^​ Infection and Immunity, Murdoch Children’s Research Institute, Royal Children’s Hospital, Parkville, Victoria, Australia; ^3^​ Department of Paediatrics, University of Melbourne, Parkville, Victoria, Australia; ^4^​ Department of Infectious Disease Epidemiology, London School of Hygiene & Tropical Medicine, London, UK; ^5^​ Department of Epidemiology of Microbial Diseases, Yale School of Public Health, New Haven, Connecticut, USA; ^6^​ Department of Microbiology and Immunology, Peter Doherty Institute for Infection and Immunity, The University of Melbourne, Parkville, Victoria, Australia; ^7^​ Institute of Medical Microbiology, Semmelweis University, Budapest, Hungary

**Keywords:** whole genome sequencing, serotyping, pneumococcus

## Abstract

Serotyping of *Streptococcus pneumoniae* is a critical tool in the surveillance of the pathogen and in the development and evaluation of vaccines. Whole-genome DNA sequencing and analysis is becoming increasingly common and is an effective method for pneumococcal serotype identification of pure isolates. However, because of the complexities of the pneumococcal capsular loci, current analysis software requires samples to be pure (or nearly pure) and only contain a single pneumococcal serotype. We introduce a new software tool called SeroCall, which can identify and quantitate the serotypes present in samples, even when several serotypes are present. The sample preparation, library preparation and sequencing follow standard laboratory protocols. The software runs as fast as or faster than existing identification tools on typical computing servers and is freely available under an open source licence at https://github.com/knightjimr/serocall. Using samples with known concentrations of different serotypes as well as blinded samples, we were able to accurately quantify the abundance of different serotypes of pneumococcus in mixed cultures, with 100 % accuracy for detecting the major serotype and up to 86 % accuracy for detecting minor serotypes. We were also able to track changes in serotype frequency over time in an experimental setting. This approach could be applied in both epidemiological field studies of pneumococcal colonization and experimental laboratory studies, and could provide a cheaper and more efficient method for serotyping than alternative approaches.

## Data Summary

The PneumoCaT serotype datasets can be accessed through the European Nucleotide Archive (ENA) under project PRJEB14267. The known mixture and replicate sample datasets can be accessed through the National Center for Biotechnology Information (NCBI) Sequence Read Archive under project PRJNA561126. The software is freely available under an open source licence at https://github.com/knightjimr/serocall.

**Impact Statement**

*
Streptococcus pneumoniae* is a bacterial pathogen responsible for causing a range of severe disease, including pneumonia, meningitis and bloodstream infections. Pneumococcus is diverse, having over 90 different serotypes that help the bacteria evade immune recognition. The serotypes are defined based on the capsular polysaccharides. A subset of the serotypes are targeted by pneumococcal conjugate vaccines. Therefore, there is a need to track changes in the epidemiology of serotypes in the population. The capsular polysaccharides themselves are encoded by genes located in a biosynthetic cassette, and the sequences of this cassette can be used to identify the serotype. A number of methods have been developed to identify the serotype of clinical samples, including next-generation sequencing. However, the current methods can either only detect a subset of the serotypes or require that the samples contain a single serotype. This is a problem for samples from the upper respiratory tract, where there are often multiple serotypes present at once. We present a whole-genome sequencing approach and software tool that is able to identify and quantitate mixed samples of multiple serotypes and that is able to identify the range of known serotypes.

## Introduction

*Streptococcus pneumoniae* (the pneumococcus) is a bacterial pathogen that causes a large burden of disease globally. Currently available protein–polysaccharide conjugate vaccines target 13 of the more than 95 identified serotypes. The vaccines reduce the frequency of colonization due to vaccine-targeted serotypes and subsequently reduce disease [1]. There is a need to perform surveillance to monitor declines in vaccine-targeted serotypes as well as to detect increases in disease caused by serotypes not targeted by the vaccine (serotype replacement). The gold standard is to monitor the incidence of invasive pneumococcal disease, a rare but severe outcome where the bacteria are isolated from a normally sterile site, such as the blood or cerebrospinal fluid. However, conducting such disease surveillance in low-resource settings is often not feasible. Therefore, it is often necessary to use other indirect measures of serotype epidemiology and vaccine effects. One such indirect measure is to track the prevalence of serotypes among healthy children who carry pneumococcus in the nasopharynx [2]. Because pneumococci are commonly detected among healthy children, point prevalence studies can be used to track changes in exposure to the different serotypes [3, 4].

Carriage-based surveillance typically involves collecting a nasopharyngeal swab from a child, culturing it in the laboratory, isolating a pneumococcal colony and then performing a traditional serotyping method such as the Quellung reaction, an antibody-based assay to determine the serotype of the isolate [5]. Quellung is relatively time-consuming to perform, particularly when trying to test multiple colonies per sample. More recently, DNA-based approaches have been used to determine the serotype of the isolated strain; for example, conventional and real-time PCR assays have been developed to identify common serotypes and/or serogroups [6–8].

Whole-genome sequencing can effectively determine the serotype of single isolates, and several pipelines (PneumoCaT and SeroBA) have been developed [9, 10]. A microarray-based platform can detect and quantify the relative abundance of all serotypes in a sample [11]. This is a highly sensitive and accurate method and outperforms many other serotyping approaches [12]. The downside of this technology is that it requires specialized equipment that cannot be readily implemented by different laboratories. An ideal solution could be a sequencing-based approach that could be used to identify multiple serotypes in mixed samples and quantify their abundance. Sequencing equipment is now widely available on standard platforms, making these methods portable and readily comparable between laboratories. Low-cost Illumina sequencing library preparation protocols make such an approach feasible and cost-effective [13], and whole-genome sequencing is increasingly being adopted for diagnostic and public health applications [14]. The major challenge is a bioinformatic one: how to accurately identify and quantify serotypes in mixed samples.

The bioinformatic challenge revolves around the similarity of portions of the sequences in the capsular biosynthesis cassette in multiple serotypes. Only 25 of the 94 serotype capsular sequences are genetically distinct, while the rest form ‘serogroups’ of genetically similar but phenotypically different serotypes [9]. The similarity is such that over 70 % of error-free reads from those groups cannot uniquely map to a specific serotype, and several phenotypically distinct serotypes differ by only a single base pair over their 10–25 kb capsular sequence. Thus, traditional read mapping approaches fail, as they assume that nearly all informative reads will map uniquely. PneumoCaT and SeroBA can accurately identify serotypes from Illumina whole-genome sequencing reads. However, they expect ‘pure’ samples (95 % or more of the sample consists of a single serotype), do not provide quantitation and will simply report ‘mixed’ if the sample is found to contain multiple serotypes. In this study we develop and test an analysis approach and a software tool, SeroCall, for quantifying serotype abundance based on raw Illumina sequencing reads. Its output will report all serotypes identified from the sample data, along with percentage estimates for each serotype. We first use existing datasets and spiked samples in the laboratory to develop and test the pipeline. We then test the performance of this approach using a reference set of blinded gold standard laboratory-prepared samples that have known quantities of different serotypes.

## Results

### Single-serotype calls using sequences in the PneumoCaT database

The development and validation datasets used to test the PneumoCaT and SeroBA software in [9, 10] were processed locally by SeroCall, PneumoCaT and SeroBA to test the concordance and speed of the SeroCall software (testing the method described in the Methods section). For the 871 development samples, the calls made by SeroCall had a concordance rate of 96.4 % (840/871) at the serotype level and 98.9 % (862/871) at the serogroup level, with 819 exactly matching calls, 21 matching calls with a low-fraction ‘minor’ call below 5 % (for example, the SeroCall output for PHESPD0784, a serotype 2 sample, was 98.3 % serotype 2 and 1.7% serotype 3), 22 samples with calls within the same serogroup but different from the determined serotype, and 9 samples that were ‘discrepant’ with any other non-matching calls.

For the 2065 validation samples, the concordance rate was 96.8 % (2000/2065) at the serotype level and 98.8 % (2041/2065) at the serogroup level, with 1923 exactly matching calls, 77 matching calls with ‘minor’ second calls, 41 samples with calls within the serogroup but different from the serotype, and 24 ‘discrepant’ samples. The details of all non-matching samples for both datasets can be found in File S2 (available in the online version of this article).

The minimum, mean and maximum execution times for SeroCall, SeroBA and PneumoCaT are given in Table 1, for the analysis of the development samples (the validation sample running times were similar). For this dataset on these servers, SeroCall ran three times faster than SeroBA and twice as fast as PneumoCaT. The computation in SeroCall is dominated by the BWA MEM alignment, which scales linearly in the number of cores. So, on compute servers with eight or more cores, SeroCall is expected to run as fast as or faster than SeroBA.

**Table 1.** Comparison of run times for SeroCall, SeroBA and PneumoCaT

Running time (MM : SS)	SeroCall	SeroBA	PneumoCaT
Minimum	0 : 14	0 : 26	0 : 17
Mean	0 : 35	1 : 43	1 : 16
Maximum	1 : 06	2 : 39	2 : 49

All software was run on 20 core, 121 GB memory, ‘2× E5-2660 v3’ computer servers, where the software was run with exclusive access to the server. The SeroCall and PneumoCaT command lines were passed ‘-t 20’ options, allowing them to use 20 parallel threads.

### All-by-all, *in silico* pairwise mixtures using different ratios

**Fig. 1.**
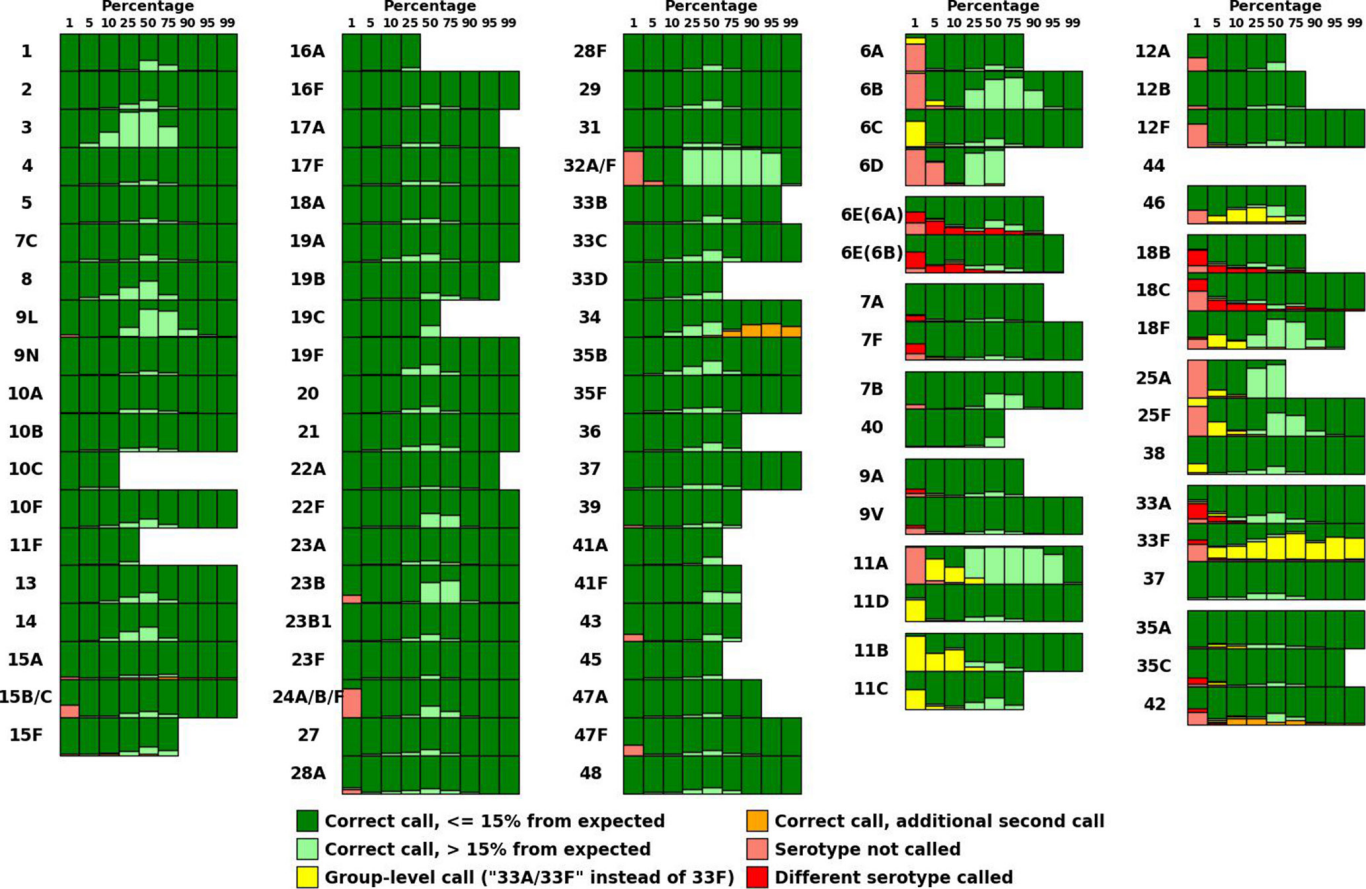
Call accuracy for all-by-all, *in silico* mixtures of 3.5 million reads. Accuracy of SeroCall for each serotype/percentage combination, displayed as vertical barcharts evaluating the calls from pairwise mixing the serotype at the given percentage against each of the other serotypes. Each call was evaluated to see if SeroCall made the correct call, did not call that serotype, had additional serotype calls or called a different serotype. Correct calls were also evaluated as to whether the reported percentage was within 15 % of that serotype’s input percentage or not. Missing (or white) barcharts reflect samples with too few reads to perform the *in silico* mixture (i.e. the serotype 10C data contained 688 206 reads, so mixtures of 3.5 million reads using percentages >=25 % could not be generated).

All pairwise mixtures of serotypes were performed using samples from the PneumoCaT database, as described in the Methods section, testing SeroCall calling at 1, 5, 10, 25, 50, 75, 90 and 95 % for each serotype (against all other serotypes), as well as testing using different numbers of sequencing reads (results using 3.5 million reads shown in Fig. 1, results for 0.5–3.0 million reads can be found in Figs S1–S6).

For most of the serotypes (the left three columns of Fig. 1), SeroCall made highly accurate calls for fractions >=5 % (58 of 59 serotypes were correctly called in all 5–99 % mixtures, with 32A/32F called in all except 7 of 52 mixtures at 5 %). SeroCall also made many accurate calls at 1 % (50 of 59 serotypes were correctly called in all mixtures at 1 %). The exceptions were that (1) one mixture of 35F at 5 % and at 10 % had an additional call of 47F at 1–2 %, and (2) four serotypes had additional, low fraction calls when mixed at a high fraction, where all additional calls were less than 1/20th of the expected serotype call fraction, with 15A/15F having extra 15B/15C calls, 34 having 33A/33F calls, 35B having 19A calls and 9L having 9A calls. An example of a low-fraction additional call is the mixture of 34 at 75 % and 13 at 25 %, where SeroCall reported serotypes 34 at 79.2 %, 13 at 20.4 % and 33F at 0.4 %.

For some serogroups, (the right two columns of Fig. 1), SeroCall has a lower rate of correct calls and a higher rate of group-level calls, additional serotype calls and different serotype calls. However, all of the additional/different calls were within the same serogroup (i.e. a 18B serotype either had an additional 18C call or was called as 18C, but had no other calls outside the serogroup). Also, excluding serotypes 6E, 18B/18C, 33F, 42 and 46, for which there is difficulty in distinguishing between members of their serogroups, SeroCall was accurate for fractions >=25 % (>99.5 % of mixture tests at 50–99 %, and 98.3 % of the mixture tests at 25 %, resulted in the correct call of the specific serotype). File S2 contains the full counts of the call evaluations and details of mixtures with additional or different calls.

The sensitivity of lower-fraction serotypes is reduced as the number of sequencing reads are reduced (as shown in Figs S1–S6). For example, at 2 million reads, only 54 of 59 serotypes not in serogroups were always called in 5 % mixtures, and only 2 of 59 were always called in 1 % mixtures. Similarly, we would expect to see an increase in sensitivity for low-fraction serotypes as the number of reads is increased.

### Mixed samples with known concentrations

**Fig. 2.**
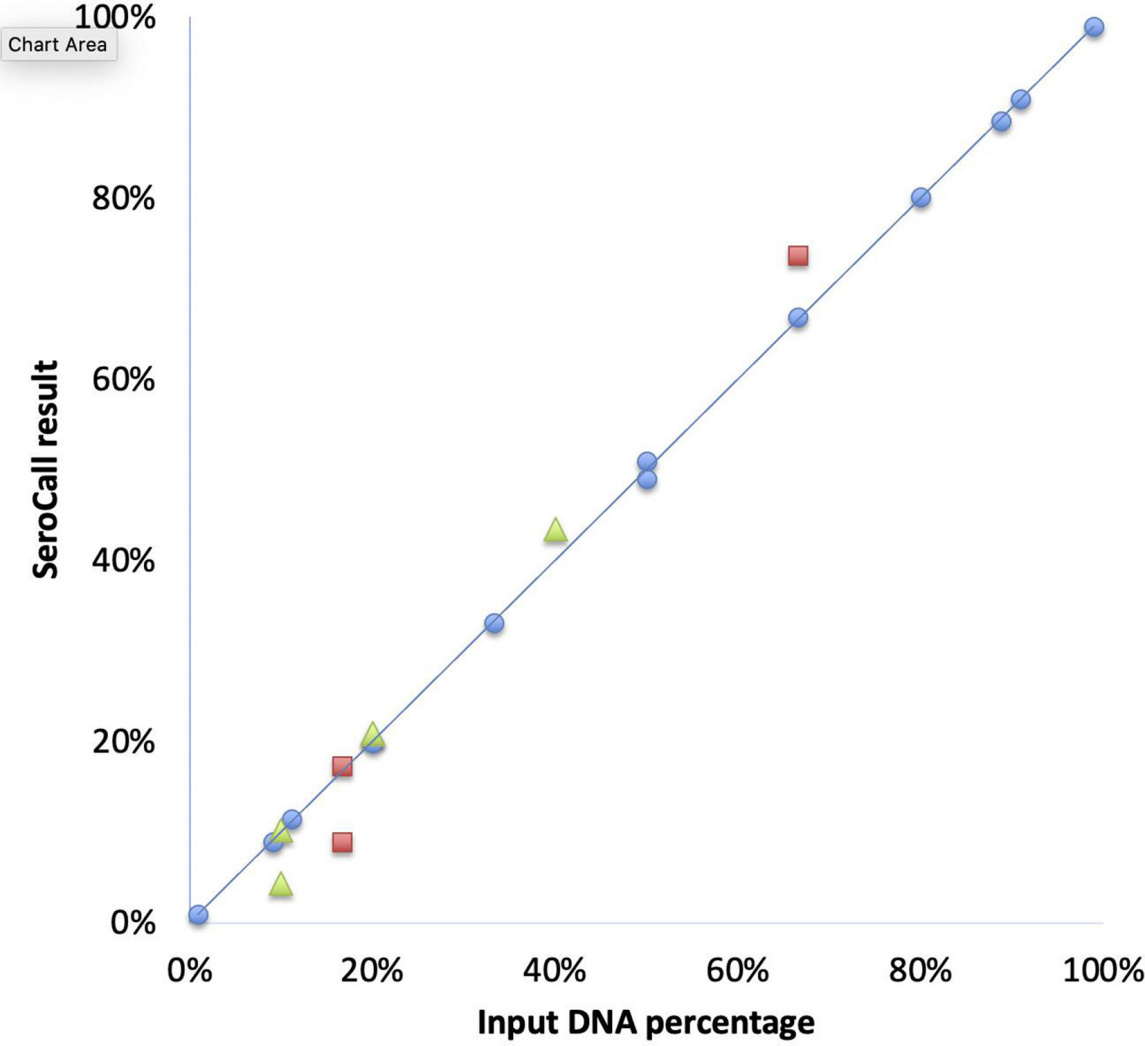
Comparison of true and estimated serotype percentage. Comparison of the true and estimated percentage of each individual serotype in multiple mixed samples using two serotypes (blue), a mixed sample with three serotypes (red) and a mixed sample with five serotypes (yellow). (Note: two 20 % serotypes from the five-serotype mix were reported as 20.9 and 21.0 %, and so overlay each other in the figure.)

Mixtures of input DNA were prepared using predetermined concentration fractions of 2, 3 and 5 different serotypes. Those mixed samples were processed, sequenced and called. SeroCall was able to accurately recover the true fraction of each serotype, including serotypes that were present at a low fraction (Fig. 2).

### Blind testing of mixed serotype samples from the PneuCarriage consortium

The PneuCarriage consortium has developed a panel of highly characterized samples, and makes available a blinded testing process for serotyping protocols, which provides the samples, analyses the serotyping results produced by the protocol, and then returns summary reports of the sensitivity and accuracy of the protocol (keeping the details of the panel blinded for future protocol testing). An 80-sample panel was provided for testing the SeroCall process and software. Fifteen of the 80 PneuCarriage samples either failed to grow (*n*=9) or had other technical issues during library preparation (*n*=6). In keeping with the blind testing, the results from all samples were returned to the PneuCarriage project and evaluated. Here we outline the results for the 65 samples that were successfully cultured, prepared and sequenced. The full evaluation of all 80 samples is detailed in Table S2.

**Fig. 3.**
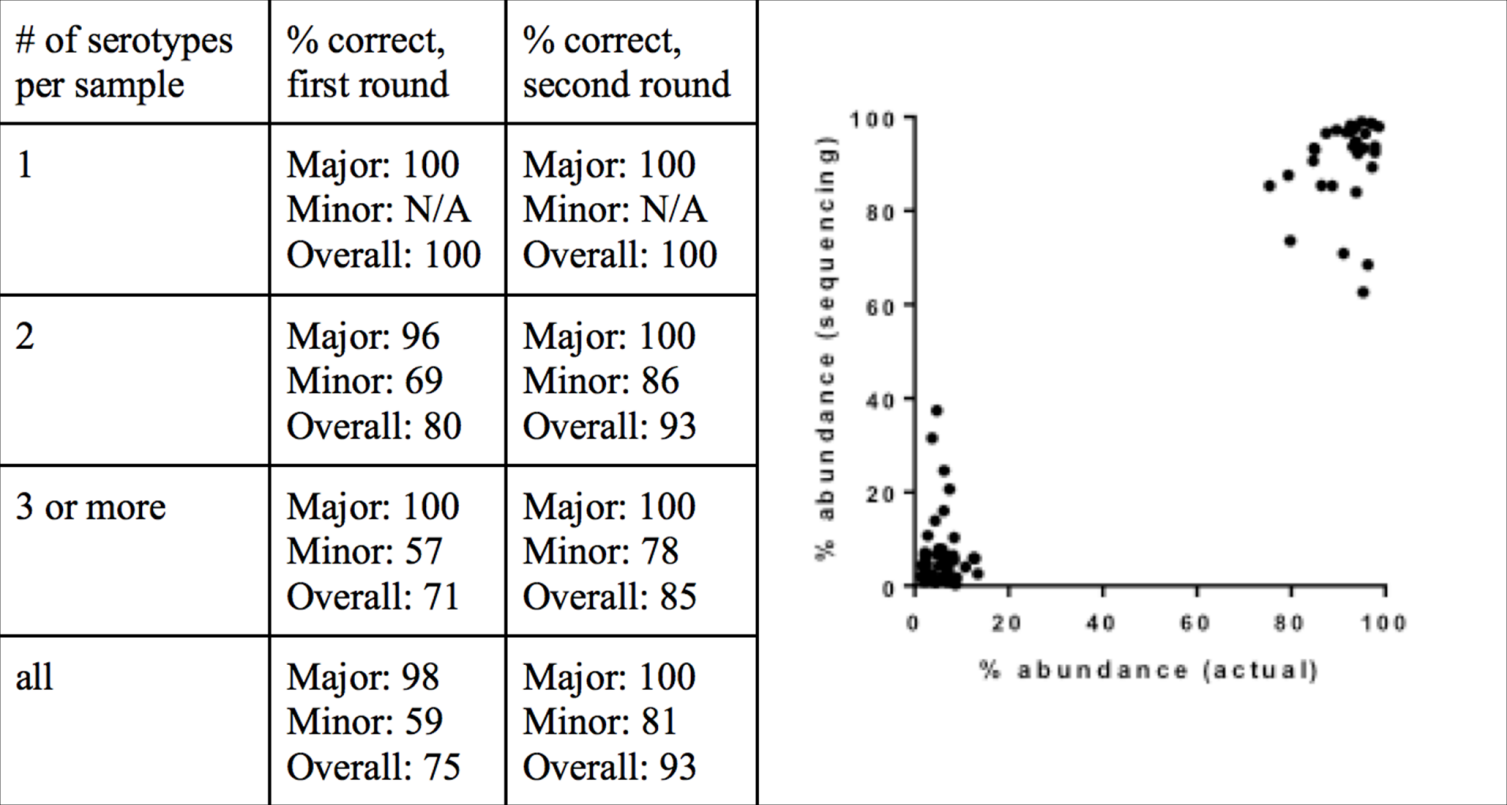
SeroCall accuracy for PneuCarriage samples. (a) Serotype calling accuracy for the 65 PneuCarriage blind testing samples, using a first round of 1.9 million reads per sample and a second round of 4.6 million reads per sample. (b) Comparison of SeroCall quantification [‘abundance (sequencing)’] and known PneuCarriage abundances [‘abundance (actual)’], for 32 mixed samples.

Fig. 3(a) shows the sensitivity of the assay, both with a first round of sequencing, and with a second round of sequencing that increased the average reads per sample. The sensitivity for detecting the major serotype was 98 and 100 % for the first and second rounds of sequencing, respectively. Samples containing serotype 12F were misidentified as 12B, as the version of SeroCall used in this testing was based on the original CTV database from the PneumoCaT paper [9]. The sensitivity for detecting minor serotypes was 59 % using only the 1.9 million reads per sample (first round), and improved to 81 % with 4.67 million reads per sample (second round). However, that came at the cost of an increase in false-positive identifications. Excluding 12F/12B misidentifications, there was one false positive in the first round [resulting in a positive predictive value (PPV) of 96 %] but six false positives in the second round (PPV of 95 %).

Finally, the quantitation of the serotype calls was evaluated against the known spiked levels for 32 multi-serotype samples called correctly (using the second round data). The correlation between the two was strong (Spearman’s *P*=0.762, *P*<0.0001), and the mean absolute difference between the known level and the SeroCall quantitation was 4.0 % (Fig. 3b).

### Longitudinal monitoring of mixed samples and replicates

**Fig. 4.**
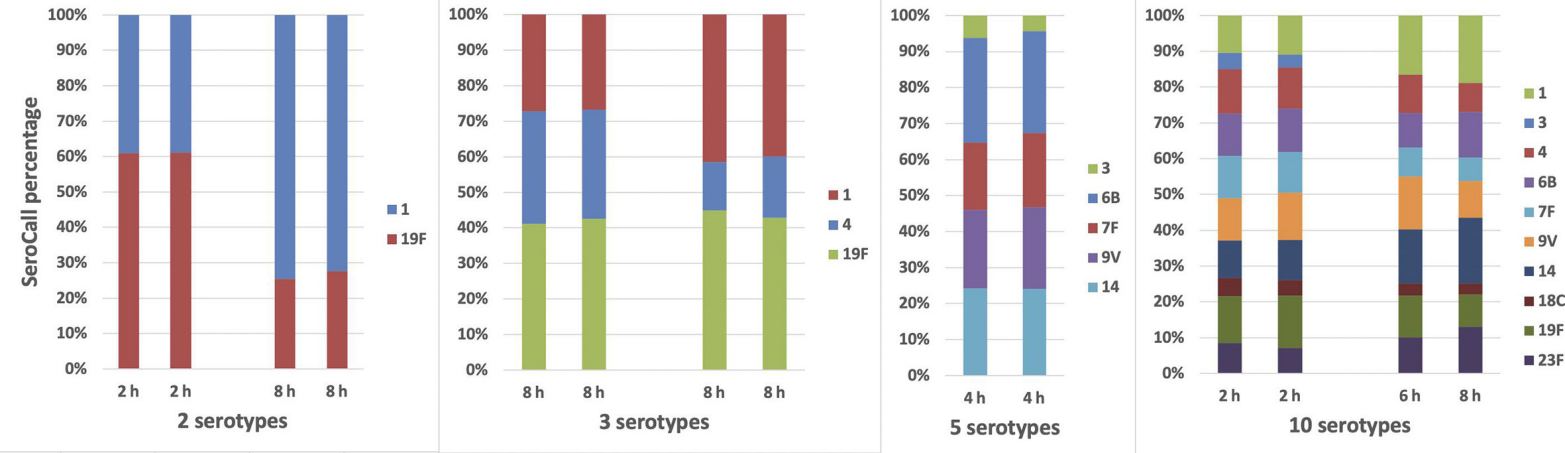
Quantitation of replicate mixtures. Replicate testing using mixtures of 2, 3, 5 and 10 serotypes. Replicates were cultured for 2, 4, 6 or 8 h before selection for sequencing.

Additionally, we sought to evaluate the ability of this approach to track changes in serotype frequency over time, approximating the setup with longitudinal carriage sampling. Mixtures of 2 to 10 clinical isolates, representing different serotypes, were grown *in vitro* (in duplicate) and sampled at 2, 4, 6 and 8 h. These replicate samples were prepared and sequenced, testing both longitudinal monitoring of changes in serotype frequency and testing reproducibility. There was good agreement between replicate samples, and it was possible to track changes in the frequency of individual serotypes over time (Fig. 4). Note, because of a primer failure, only single results were generated for the 10-serotype sample at 6 and 8 h.

## Discussion

We developed and validated the whole-genome sequencing method and analysis software SeroCall for the identification and quantification of *S. pneumoniae *. The software was tested using both internally and externally generated samples and datasets, and returned concordant results with other serotyping methodologies and other whole-genome sequencing-based serotype identification software. It also showed good agreement between replicates in tracking changes in the proportion of serotypes over time in mixed cultures in an experimental setting. SeroCall is the first sequencing-based method to perform serotype quantitation on mixed samples. Its computational performance matches or exceeds that of other software, and could be implemented efficiently to enable high-throughput surveillance of population serotypes.

Antibody-based serotyping (Quellung and latex agglutination kits) are easy and quick to use when checking the serotype of one colony. However, understanding multiple carriage is difficult with Quellung [15]. Scaling to a large number of samples can also be labour-intensive with antibody-based methods. The existing DNA-based methods have insufficient sensitivity or specificity to identify multiple serotypes, or are microarray-based. Compared to microarrays, access to/use of sequencing is increasing for public health surveillance, and costs are going down. Hence implementing carriage surveillance with a low-cost sequence-based multiple carriage surveillance is timely.

Our simulation analysis demonstrates the strong performance of this approach as well as the limitations. There are certain serotypes that are difficult to distinguish from other members of the serogroup, and this is particularly an issue when the minority serotype is at a low frequency. Performance of the method also clearly depends on read depth and will be less accurate with lower depth. Therefore results from samples with low-frequency serotype calls or low read depth should be interpreted with caution and validated with other methods.

While the method was concordant with other methodologies, there are still some limitations. As for all serotyping methods, upstream aspects, including sample storage and culture, remain important; this is reflected in the fact that we could not obtain results from nine culture-negative samples from the PneuCarriage set. The *in vitro* culture step prior to DNA extraction, as well as the DNA extraction protocol itself, could influence the detected proportion of serotypes. The quantification methods use the current set of capsular sequences as a core ‘truth set’ from which to compare the sequencing results. If there are CPS biosynthesis loci recombinations that are not present in the current dataset, serotypes might be misquantified or misclassified. SeroCall uses the entire genetic sequence to perform its classifications and quantifications, and for samples where the phenotypic serotype differs from the overall genetic serotype ancestry, misclassifications may occur. Also, complex mixtures of very closely related serotypes may be more difficult to quantify accurately than more genetically distinct serotypes. Additionally, non-pneumococcal streptococci present in the nasopharynx can confound sequenced-based serotyping [16]. As such, future work will evaluate SeroCall using nasopharyngeal samples, for example the PneuCarriage field samples (aliquots of STGG from NP swabs). Finally, improvements and increased testing of the laboratory methodologies, including the use of non-duplicate Illumina index primers and potentially side-by-side comparison of microarray and sequencing of the same DNA, to explore differences in DNA extraction efficiency, may increase the robustness of the method.

One experimental parameter that affects the results is the read depth. Using the blinded samples, we found that with a low read depth, the sensitivity for detecting minor serotypes was greatly reduced. We recommend obtained a read depth of 2–3 million reads per sample to obtain similar sensitivity to that reported here, and possibly higher depth if looking for very low-abundance serotypes. The ‘cost’ of increased read depth was an increase in the number of false-positive identifications of rare serotypes. One possible explanation for the false positives is Illumina ‘barcode hopping’ [17], as the library preparation used standard multiplex primer sequences that have been found to be susceptible to that. Using unique barcode pairs for each sample could help to avoid this issue and allow for the detection of low-abundance serotypes without an increase in false positivity.

The library preparation protocol that we use, which was developed by Baym *et al*. [13], can produce high-quality, low-cost sequences when multiplexing samples. Provided that an investigator has access to an Illumina sequencer, this makes performing sequence-based serotyping cost-effective compared with traditional serotyping methods.

During sample preparation we lost several samples. Nine of the blinded samples failed to culture. This could have been an issue with sample transport or with the culture conditions in the laboratory. We also lost several samples due to a primer failure during the library preparation. Confirming the concentration of each sample prior to pooling would catch this issue sufficiently early to allow for regeneration of the libraries for the affected samples.

In conclusion, we describe the development of an analytical tool that can be used to quantify the abundance of multiple serotypes in mixed cultures using a sequencing-based approach. We do this by addressing a bioinformatic challenge in assigning Illumina reads from a mixed sample to the correct serotype. This method could be applied to epidemiological studies of pneumococcal carriage that seek to evaluate the carriage frequency of dominant and sub-dominant serotypes and can be used to monitor changes associated with the introduction of conjugate vaccines.

## Methods

### Experiments with known serotype composition

The first set of dilution and competition experiments used invasive pneumococcal disease isolates that were obtained from the Centers for Disease Control and Prevention’s (CDC’s) Active Bacterial Core surveillance system isolate bank (1, 3, 4, 6B, 7F, 9V, 14, 18C, 19F, 23F). The strains were grown overnight on TSAII plates with 5 % sheep’s blood at 37 °C with 5 % CO_2_.

### Mixture with known concentrations of DNA

Overnight growth was harvested in phosphate-buffered saline (PBS), and genomic DNA was extracted using a DNEasy blood and tissue kit (Qiagen) with the Gram-positive pretreatment protocol. DNA was quantified using a NanoDrop reader (Thermo Fisher). Serotypes were then mixed together at the following ratios: 19F : 23F [1 : 2], 19F:23F [1 : 4], 19F:23F [1 : 8], 19F:23F [1 : 10], 19F:23F [1 : 100], 19F:23F:1 [1 : 4 : 1], 19F:23F:1 : 4 : 18C [1 : 2 : 4 : 2 : 1]. These samples were sequenced to an average of 2.76 million reads per sample on an Illumina HiSeq.

### Longitudinal growth experiment

Overnight growth on TSAII plates was resuspended in PBS, and the optical density (OD) at 600 nm was adjusted to 0.05. These stocks were then diluted 1 : 20 into a diluted broth of 7.5 ml PBS, 2.5 ml brain heart infusion (BHI), 8.25 μl sheep blood, and 125 μl horse serum. The strains were grown individually for several hours until the lowest concentration strain reached OD ~0.15. All strains were adjusted down to match this value. The strains were then mixed at equal concentration and diluted 1 : 20 into fresh broth in deep-well plates (800 μl broth+40 μl bacteria) with a separate replicate well for each time point. The plate was then incubated at 37 °C with 5 % CO_2_. Forty microlitres from each well of the 6 h time point was used to seed a new row of 800 μl broth to allow another 2 h of growth. This passaging step is important because in the limited-nutrient broth, pneumococcal population tends to crash after 6 h. At the indicated time points, the full volume of the well was transferred to the −80 °C freezer. At the end of the experiment, DNA from all wells was extracted at the same time using a Qiagen DNEasy blood and tissue kit with the Gram-positive pretreatment protocol (Qiagen). The experiment was performed in duplicate.

### Single-serotype, *in silico* mixture testing and benchmarking against other serotyping software

The development and validation whole-genome sequencing datasets that were used previously [9] to develop and test the PneumoCaT and SeroBA software were used similarly here. Eight hundred and seventy-one development samples, covering all 94 serotypes, were used in the development of the SeroCall software, and then the 2065 validation samples covering 72 serotypes were evaluated using the final version of the software. Also, the current versions of PneumoCaT (v1.2) and SeroBA (v1.0.1) were run locally on all samples, and the ‘gold standard’ calls used for benchmarking were the majority vote of the original laboratory serotyping, the PneumoCaT call and the SeroBA call. This accounts for updates to the software that correct for serotypes reported at the time of the PneumoCaT publications (most notably, a change in the 12B vs 12F typing that was discovered after the publication of [9]).

For the all-by-all, *in silico* mixture testing, a sample for each serotype was chosen from the PneumoCaT database, based on the sample with the largest number of reads (to ensure the most mixtures could be tested), where all four testing methods (SeroCall, PneumoCaT, SeroBA and serotyping) identified the same serotype for the sample. For four serotypes, no sample was called the same by all four methods, and the following samples were selected for three of those: PHESPV1446 was chosen for serotype 7A, as it was called as 7A by SeroCall, PneumoCaT and serotyping (SeroBA called it as mixed 7A/7F); PHESPD0013 was chosen for 11D, as it was called as 11D by SeroCall, SeroBA and serotyping (PneumoCaT called it as 11A); PHESPD0234 was chosen for 33A, as it was called as 33A by SeroCall, SeroBA and serotyping (PneumoCaT called it as 33F). No sample with sufficiently concordant calls was made for serotype 44, so it was not used in the testing. File S2 lists the samples chosen for each serotype along with the number of reads in each sample’s data and the percentage of those reads that align to any of the capsular sequences.

All pairs of serotypes were mixed at percentages of 1, 5, 10, 25, 50, 75, 90, 95 and 99 % (with the reciprocal percentage tested for the other serotype in the pair) and by randomly selecting reads to generate 0.5, 1.0, 1.5, 2.0, 2.5, 3.0 and 3.5 million reads of the appropriate mixture. However, because the percentages of capsular sequences to the number of reads was different for different samples (possibly due to genomic/plasmid differences between samples, and which would skew the *in silico* mixture ratios and affect the ability to distinguish sample fluctuations from software quantification errors), the random selection ensured that the ratio of capsular reads matched the percentages for the pair, instead of the ratio of total reads. The resulting datasets of over 250 000 mixtures were processed by SeroCall.

### Evaluation with blinded samples

The PneuCarriage Project [12] was a multi-centre study to evaluate pneumococcal serotyping methods. A set of standard laboratory-prepared sample mixtures has been evaluated using a large number of serotyping methods. Eighty of these samples, containing mixtures of 0–4 serotypes, were evaluated using our analysis pipeline. The laboratory personnel processing the samples and the bioinformatic analysts were blinded to the serotype composition of the samples.

The samples were provided as frozen aliquots. Samples were thawed and 10-fold dilutions were spread on a TSAII plate with 5 % sheep’s blood and incubated overnight at 37 °C with 5 % CO_2_. The most concentrated non-confluent dilution was harvested into PBS, and DNA was extracted as described above. During the culturing and preparation, nine of the serotype-positive samples failed to culture, and a further six samples failed amplification due to an existing primer failure. The remaining 65 samples were sequenced to an average of 1.90 million reads per sample; a second round of sequencing increased the average reads to 4.67 million per sample. Serotype calls and quantifications were returned for all samples and evaluated by PneuCarriage Project personnel.

### Library preparation

Illumina libraries were prepared using the protocol described by Baym *et. al*. [13]. The exception was that the final cleanup was performed using Qiagen PCR purification columns rather than the bead-based assay described in the original paper. The sequencing indices that were used are listed in Table S3. Each plate of up to 96 samples was multiplexed and run on an Illumina HiSeq with a read length of 2×150 bp.

### Bioinformatic approach

The overall steps of the SeroCall software follow those of PneumoCaT and SeroBA, but apply different algorithms in order to quantify all serotypes found in a sample. All of the tools (1) align the read data to the set of serotype capsular sequences, (2) identify serogroups and distinct serotypes that are present in the sample and (3) distinguish serotypes within the serogroups, using serotype-specific variants or regions of the capsular sequences.

#### Step 1 – read alignments

Sequence read data are first aligned using BWA MEM [18] to a ‘reference’ that combines the serotype capsular sequences from 94 serotypes with the non-capsular sequences from 3 *S. pneumoniae* genomes: R6, SPNA45 and ATCC700669 (where the capsular sequence from each has been masked). File S1 details the capsular reference sequences used, most of which are the same as the PneumoCaT reference sequences, but several have been modified to work with this algorithm. The genome sequences mainly serve as a ‘decoy’, so that genomic reads will align to the genome sequences instead of to the capsular sequences, and so will not affect the read depths for the serotypes.

The read alignments produced by BWA MEM are used to compute ‘bin counts,’ counting the total and uniquely mapped reads across the serotype capsular sequences. Each sequence is partitioned into 500 bp bins, denoted *S*_*i,b*_ for serotype *i* and bin *b*. The choice of 500 bp ensures that local depth variations resulting from sequencing are smoothed in the bin counts. Read alignments are processed in read pairs, and are first filtered for (1) any genomic alignments, (2) any unmapped alignments (if either read in a read pair is unaligned, then both reads in the pair are filtered), (3) any chimeric reads aligning to two different serotypes, or (4) any read pairs with a combined 10 or more differing or soft clipped bases, as this is a sign of a genomic read pair mistakenly aligned to a serotype sequence.

The remaining read pair alignments add to bin counts, incrementing the counts of any bin which overlaps with either of the read alignments. The ‘total bin counts’ count both uniquely mapping reads (reads whose MQ >=0) and repetitively mapping reads (could map equally well to multiple locations in the serotype sequences, where the BWA MEM software randomly chooses a location from those best locations). The ‘unique bin counts’ only count uniquely mapping reads. For an input sequence dataset, this results in *T*_*i,b*_ and *U*_*i,b*_ matrices containing the bin counts for that data.

#### Step 2 – serotype/serogroup quantification

The second step takes the bin counts from the input data, treating them as the ‘observed’ counts *OT*_*i,b*_ and *OU*_*i,b*_, and compares them against the sets of ‘expected’ counts *ET*_*y,i,b*_ and *EU*_*y,i,b*_ for all serotypes *y* (because of the similarity between serotype sequences, reads from a serotype *y* will align to serotype *i*, and so will contribute to the bin counts for serotype *i*). These expected counts were determined by generating simulated reads for each serotype and computing the bin counts for those reads. Specifically, a simulated 2×100 bp read pair (with an insert size of 200 bp) was generated at every position of a serotype’s capsular sequence, so that each location of the capsular sequence is covered by 200 reads across the whole sequence, except at the ends. Generating bin counts in this way results in expected bin counts for an equal 200x sampling of each serotype.

The comparison involves an optimization computation using gradient descent, to compute ‘factor levels’ *F*_*y*_, for each serotype *y*, which optimize the equations:

$$OT_{i,b}≅\sum_{y}(ET_{y,i,b}*F_{y})$$

$$OU_{i,b}≅\sum_{y}(EU_{y,i,b}*F_{y})$$

In other words, it computes the factor levels that result in a mixture of the expected serotype bin counts that most closely resembles the observed bin counts. Initially, all *F*_*y*_ are set to 1.0, and then 100 rounds of a gradient descent algorithm are performed, computing the expected mixture bin counts, comparing them to the observed counts and adjusting the factor levels up or down.

Specifically, each round of the algorithm first computes:

$$MT_{i,b}≅\sum_{y}\left(ET_{y,i,b}*F_{y}\right)$$

$$MU_{i,b}≅\sum_{y}\left(EU_{y,i,b}*F_{y}\right)$$

using the current *F*_*y*_ values. Then the algorithm computes bin ratios *RT*_*i,b*_=*OT*_*i,b*_/*MT*_*i,b*_ and *RU*_*i,b*_=*OU*_*i,b*_/*MU*_*i,b*_. Ideally, all of the ratios should equal 1.0, if the computed mixture matches the observed counts across all of the bins. Per-serotype factor ratios, *ratioT*_*i*_ and *ratioU*_*i*_, are then computed using a weighted median of the bin ratios for the serotype, where the weights for each bin are *ET*_*i,i,b*_/*maxET* and *EU*_*i,i,b*_/*maxEU* (i.e. the fraction of total/unique reads coming from serotype *i* and bin *b* that were actually counted in the bin). This gives higher weight to the more unique, or less repetitive, regions of each serotype’s capsular sequence. And the median is used instead of the mean in order to prevent genome contamination and local genetic differences (serotype samples whose actual capsular sequence mainly matches the reference, but contains a local region unique to another serotype) from skewing the quantitation.

Final serotype ratios are computed by combining the *ratioT* and *ratioU* values based on the bin with the highest unique weight:

$$MW_{i}=\max_{b}\left(\frac{EU_{i,i,b}}{maxEU}\right)$$

$$ratio_{i}=MW_{i}*ratioU_{i}+\left(1.0-MW_{i}\right)*ratioT_{i}$$

New factor levels *F*_*y*_′ are computed as *F*_*y*_′=*F*_*y*_+(*F*_*y*_ *−F*_y_* *ratio*_*y*_)/2, adjusting the factor level by half of the computed observed over expected ratio, in each round of the gradient descent. Also, if at any point, *F*_*y*_′ falls below 0.002, it is set to 0.0 (this threshold is a computational speed optimization, to stop evaluating samples whose factors fall far below the 1 % fraction range, but have not yet reached 0.0 through the descent process). The new *F*_*y*_′ values then become the factor levels *F*_*y*_ in the next round of the computation.

#### Step 3 – serogroup refinement

The third and final step readjusts the factor levels for the serotypes within serogroups that cannot be distinguished based on bin-sized read depth differences. The methods of steps 1 and 2 are able to distinguish 56 of the serotypes without further refinement, and the groups of serotypes that require further refinement by this method are: 6A/6B/6C/6D/6E; 7A/7F; 7B/40; 9A/9V; 11A/11D; 11B/11C; 12A/12B/12F/44/46; 15B/15C; 18B/18C/18F; 24B/24F; 25A/25F/38; 32A/32F; 33A/33F/37; 35A/35C/42.

Serotypes 15B and 15C can interconvert [19] and so are reported as 15B/15C. In keeping with the reporting performed by PneumoCaT, serogroups 24 and 32 are reported at the group level. For the other groups, the CTV database in PneumoCaT was used to identify variants and genes/alleles that differ between serotypes in the groups. Each of those differences was translated into the sets of locations within the full capsular sequence references, instead of the CTV database’s reporting of a gene position or gene sequence. For example, the CTV database identifies a SNP in *wcjE* at position 721 where serotypes 9A and 9V are different. This is translated into the locations 18 534 and 18 852 in the 9A and 9V capsular sequences, respectively. The list of all differences used is given in the Table S1.

The reason for the translation is that this step of the algorithm takes advantage of the sensitivity of BWA MEM in aligning reads to near-identical locations in the reference. If there is a single location whose alignment contains more identities than all other locations, that ‘best match’ location will be chosen. Even a single nucleotide difference is sufficient for BWA MEM to consistently align reads to the proper serotype’s sequence, and so the read depths at those difference locations provide an accurate measure of the differences between serotypes. So, instead of performing a separate variant calling, mapping or *de novo* assembly to resolve serotypes, the computation of step 1 computes ‘bin’ counts at these specific difference locations, and then this step compares the observed counts at those locations against the expected counts.

Since these locations are where the serotypes are genetically different, and alignment ‘bleed’ is not an issue, this step just computes depth ratios for each serotype and difference location in a group, where OU/EU is used if EU is greater than 0, and OT/ET is used otherwise. The ratio for a serotype is the minimum of the computed depth ratios across the difference locations (if the serotype is present in the sample, each of these locations should have a non-zero read depth). Then, those ratios are summed, and serotype percentages are computed by dividing the serotype ratio by the sum of the ratios.

If the sum of the ratios is 0, this means that there is no read evidence distinguishing the serotypes in the group, and an ambiguous call like ‘09A/09V’ is made, with a factor level equal to the sum of the factor levels computed in step 2, for the serotypes in the group. If the sum is greater than 0, then the factor levels from step 2 (again, for the serotypes in the group) are reapportioned using the serotype percentages computed in this step. So, for example, if 09A and 09V had step 2 factor levels of 0.13 and 0.11, but the step 3 serotype percentages were 80 % 09A and 20 % 09V, then the factor levels would be changed to 0.192 for 09A and 0.048 for 09V (to maintain the 09A/09V levels compared to all other serotypes, but reset the serotype-specific levels to the identified percentages).

Once the final factor levels are computed, they are converted to percentages by dividing each by the sum of all factor levels. Then, any serotype with a percentage less than 0.2 % is filtered out, and the percentages are recomputed using only the remaining serotypes. Those serotypes and percentages form the output calls produced by the software.

### Data and software availability

The PneumoCaT serotype datasets can be accessed through the European Nucleotide Archive (ENA) under project PRJEB14267. The known mixture and replicate sample datasets can be accessed through the National Center for Biotechnology Information (NCBI) Sequence Read Archive under project PRJNA561126. The software is freely available under an open source licence at https://github.com/knightjimr/serocall.

## Supplementary Data

Supplementary material 1

Supplementary material 2
