# Supplementary material for: Determining the serotype composition of mixed samples of pneumococcus using whole-genome sequencing
Source: Microb Genom. 2020 Dec 23;7(1):mgen000494. doi: 10.1099/mgen.0.000494 (PMC8115901; doi:10.1099/mgen.0.000494)
Supplement: Supplementary material 1 [file mgen-7-494-s001.pdf]

Appendix

Reference sequences used by SeroCall

The capsular sequences from PneumoCaT's v1.2 streptococcus-pneumoniae-ctvdb database were used as the reference capsular sequences for SeroCall, with the following alterations:

- PneumoCaT's 06E sequence was duplicated into 06E_6A and 06E_6B, and then the 06E_6A sequence was edited, changing base 7912 from A to G (to match the difference used to distinguish 6A and 6B, see below).
- PneumoCaT's 25A sequence was edited so that the *wcyC* sequence matches the *wcyC*_25A sequence found in 25A_25F_38/reference.fasta. The reason for this is that the PneumoCaT 25A sequence, and the original 25A reference from [1] (accession CR931689.2), contain the *wcyC*_25F sequence, not *wcyC*_25A. (This does not affect PneumoCaT or SeroBA, because they use the separate gene sequences in the second phase of their analysis. However, SeroCall uses only the primary reference, so it requires the correct 25A *wcyC* sequence to be there.)
- PneumoCaT's 33F sequence was extended with the rest of the sequence from [1] (accession CR931702.1), starting at base 1205 of that sequence, so as to restore the presence of the *wcjE* gene in the capsular sequence. (The 14,496 bp PneumoCaT sequence exactly matches CR931702.1 from base 1205..15700, but does not contain the last 1,299 bases of CR931702.1.)
- PneumoCaT's 37 sequence, which consists only of the tts gene sequence, was replaced with the sequence from [1] (accession CR931709.1) starting at base 1587, which is the location matching the beginning of the PneumoCaT 33A and 33F sequences. Then, that sequence was appended by 40 N's and the *tts* gene sequence. This was done so that reads

from the non-functional 37 capsular sequence are aligned to this sequence, instead of being aligned to the 33A and 33F sequences.

The *S. pneumoniae* genome sequences used as decoy sequence for read alignments are the R6 reference (accession NC_003098.1), SPNA45 reference (accession HE983624.1) and ATCC700669 reference (accession FM211187.1).

Sequence differences used to distinguish serogroup members

In phase 3 of the SeroCall algorithm, sets of locations are used to distinguish the presence of specific serotypes that cannot be distinguished by larger genetic differences in phase 2. The following difference locations are used:

**ID**	**Serotype:Location**				
diff1	06A:7577	06B:7577	06C:7384	06D:7384	
diff2	06A:5400-5500	06B:5400-5500	06C:5400-5500	06D:5400-5500	
diff3	06E(6A):7850	06E(6B):7850			
diff4	07A:8598	07F:8582			
diff5	07B:8250	40:8259			
diff6	07B:8590	40:8600			
diff7	09A:16996	09V:16996			
diff8	11A:8237	11D:8237			
diff9	11B:13140	11C:13122			
diff10	12A:4900-5000	12B:4200-4300	12F:4200-4300	44:4200-4300	46:4900-5000
diff11	12A:8077	12B:7195	12F:7204	44:7204	46:8077
diff12	12A:9822	12B:8940	12F:8949	44:8949	46:9822
diff13	12A:12004	12B:11122	12F:11131	44:11131	46:12004
diff14	12A:12217	12B:11335	12F:11344	44:11344	46:12217
diff15	18B:7700-7800	18C:7700-7800	18F:7700-7800		
diff16	18B:12382	18C:12382	18F:12382		

diff17	25A:11800-11900	25F:11800-11900	38:10000-101100		
diff18	25A:10477	25F:10477	38:8645		
diff19	28A:7049-7080	28F:7049-7080			
diff20	33A:14968	33F:14968	37:16400-16500		
diff21	35A:10753	35C:10758	42:10758		
diff22	35A:1973	35C:1982	42:1982		

Table S1. Reference sequence differences to distinguish serogroup serotypes.

In-silico mixture results at different sequencing levels

The following figures summaries the all-by-all, in-silico mixture testing results as the number of sequencing reads sampled for the mixtures is reduced. Each figure shows the results of serotype/percentage mixture tests against all other serotypes, displayed as a vertical barchart. Any missing barchart reflects a serotype's data that contained too few reads to perform the mixture test.

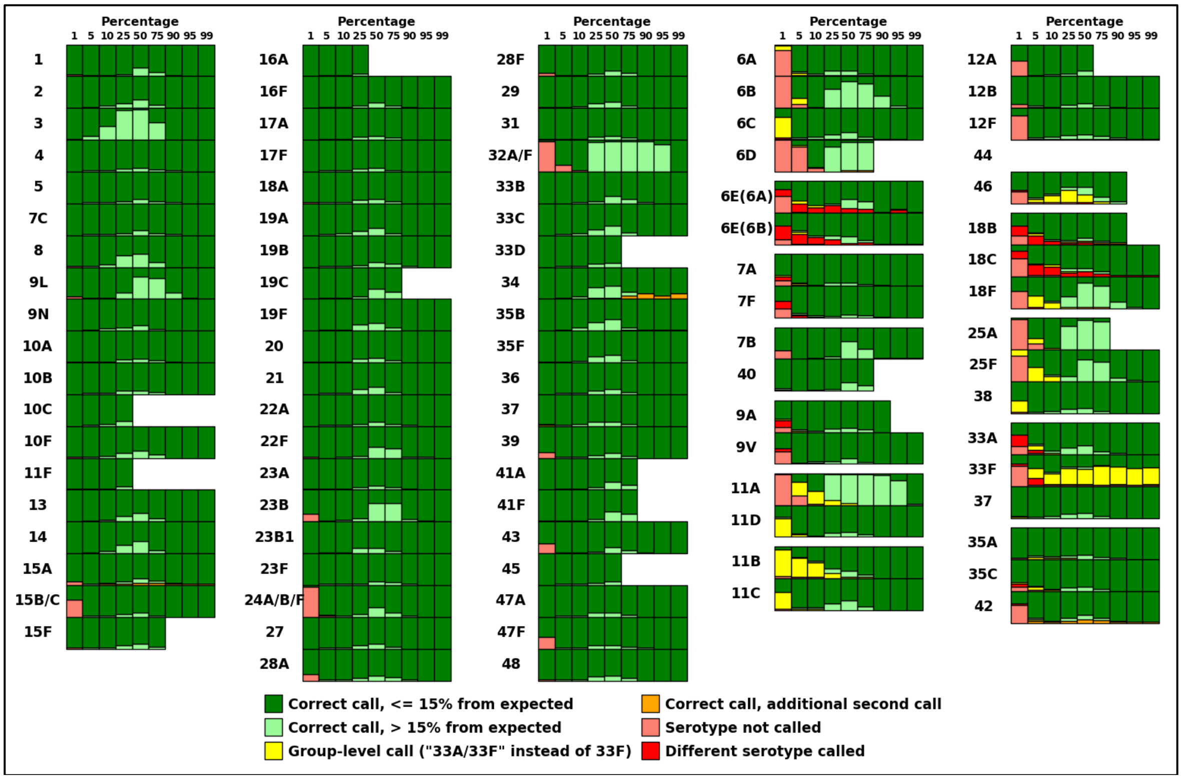

Figure S1. In-silico mixture results for 3.0 million reads.

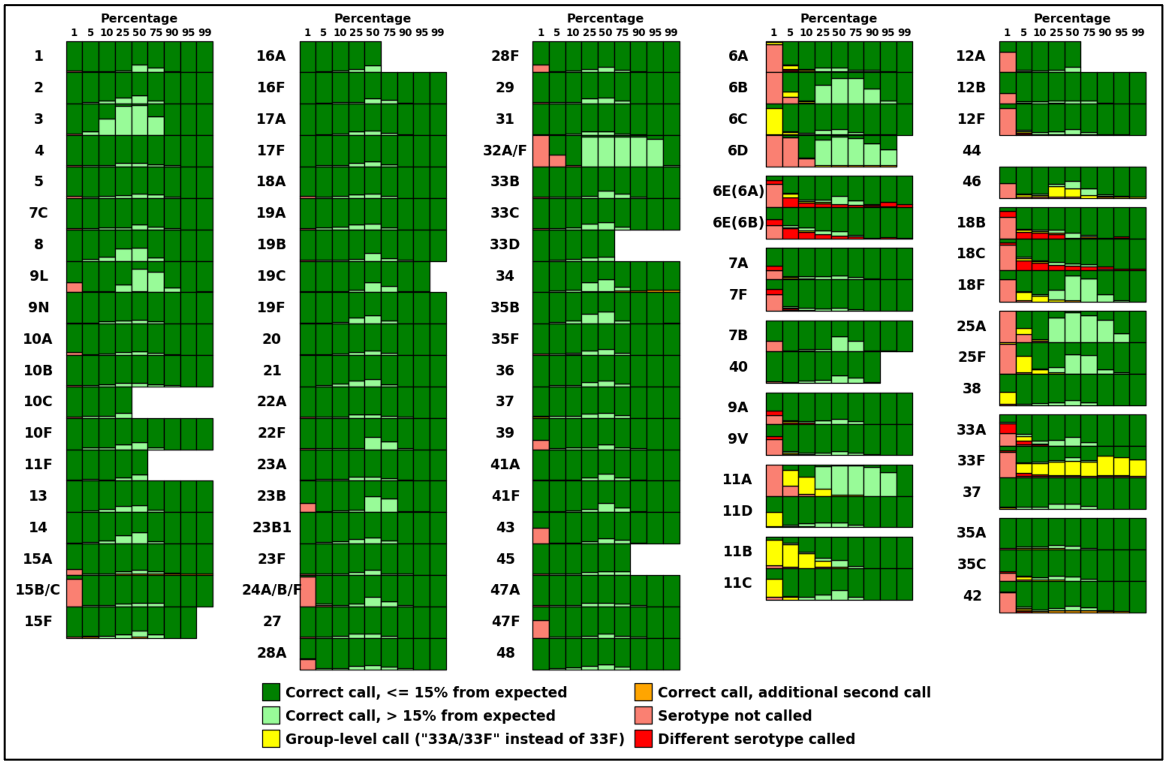

Figure S2. In-silico mixture results for 2.5 million reads.

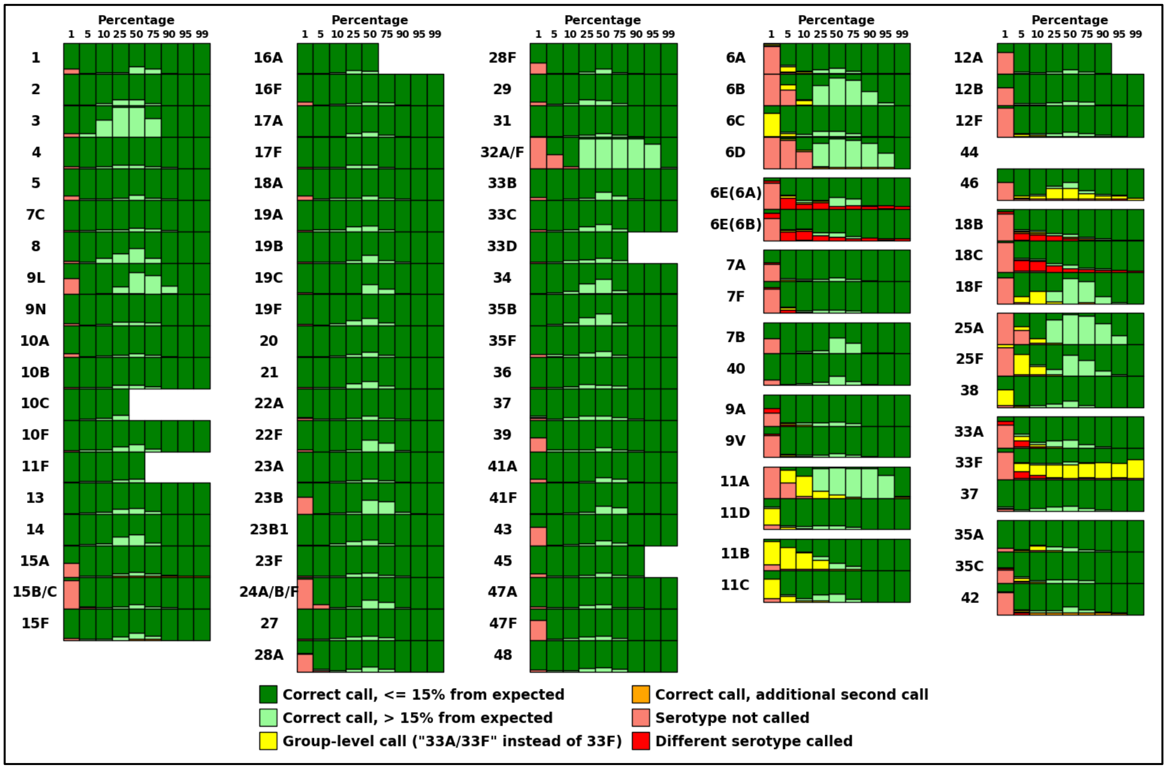

Figure S3. In-silico mixture results for 2.0 million reads.

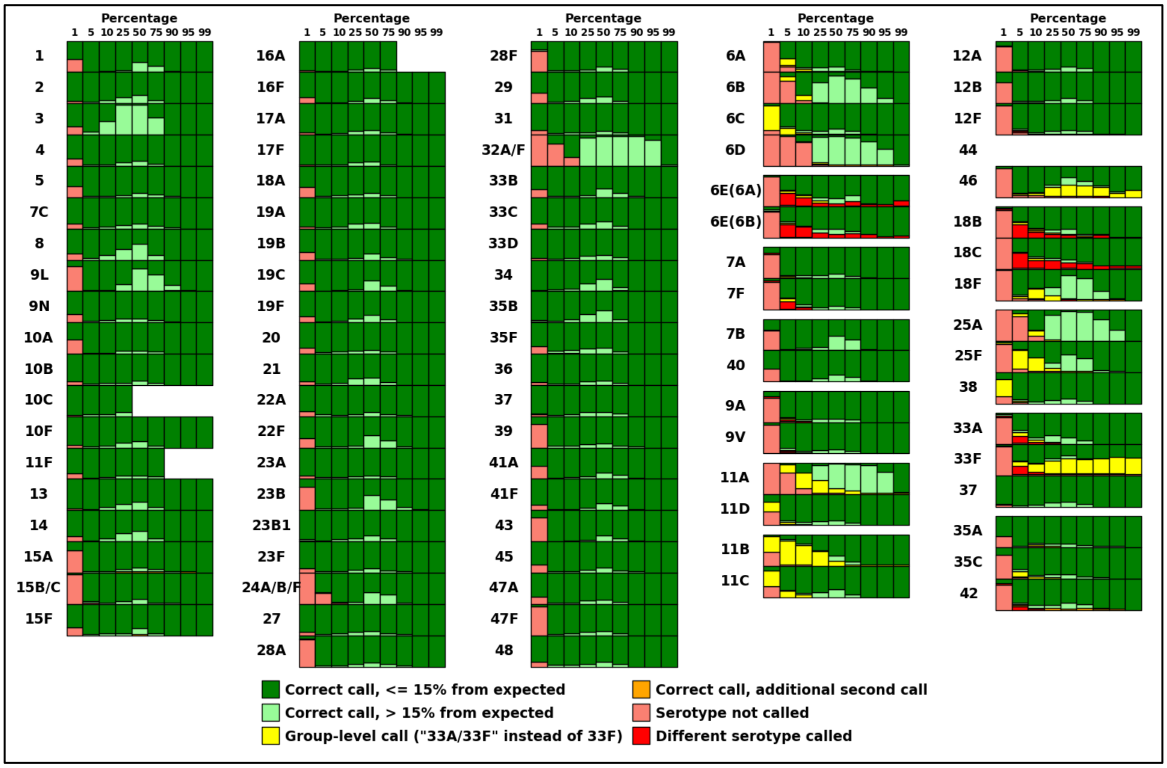

Figure S4. In-silico mixture results for 1.5 million reads.

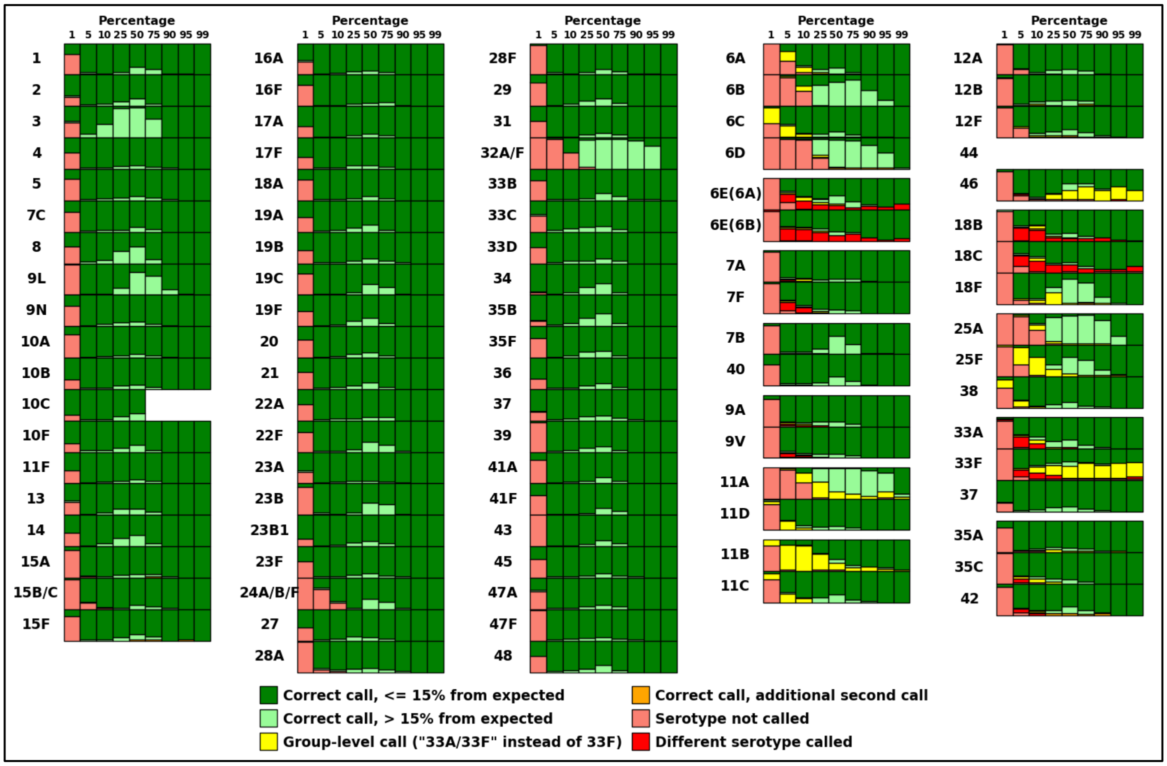

Figure S5. In-silico mixture results for 1.0 million reads.

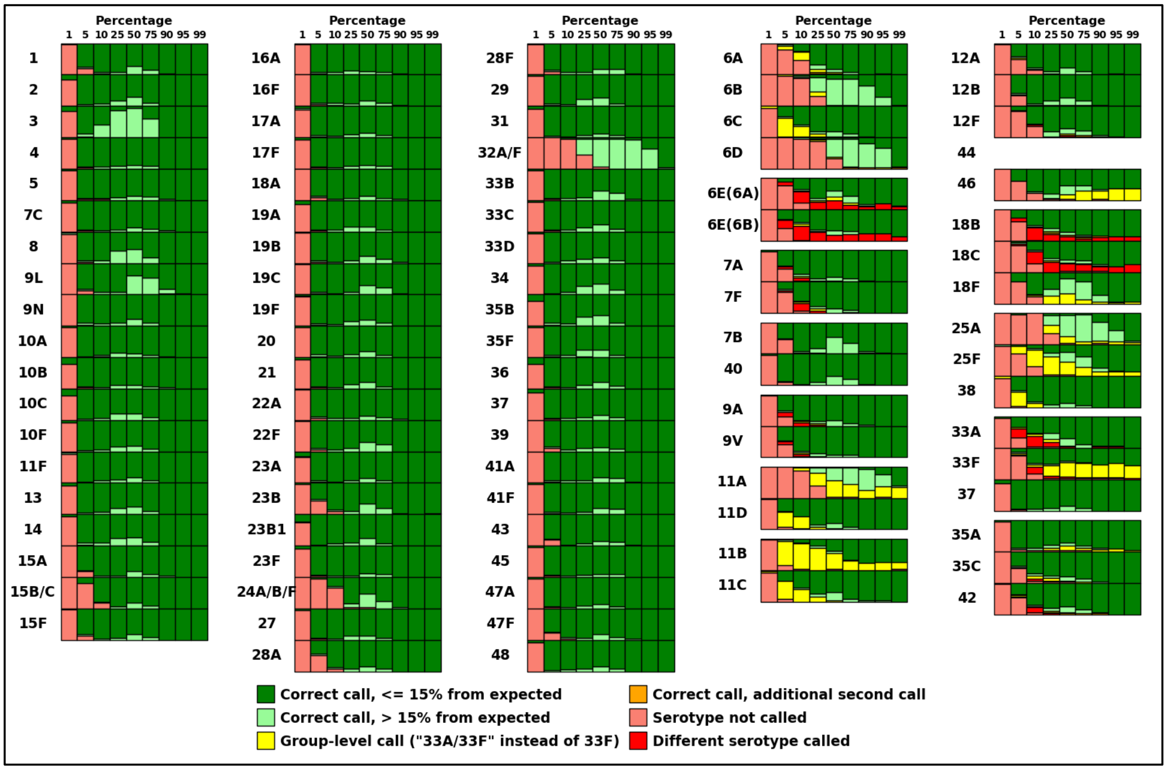

Figure S6. In-silico mixture results for 0.5 million reads.

Full sensitivity table from the PneuCarriage report

Following the reporting standards used by the PneuCarriage project, all samples tested were used to determine the sensitivity and specificity of the serotyping method. This included samples that were successfully sequenced as well as those that failed to culture and those that had other sample preparation failures. The table below was taken directly from the PneuCarriage report, giving the full sensitivity metrics (1) for all samples, (2) for all samples except the 9 culture negative samples, and (3) for all samples except the culture negative and primer failure samples. Also, the table reports results from both rounds of evaluation, where the first round occurred after one round of sequencing (with an average of 1.9 million reads per sample), and the second occurred after a second round of sequencing was performed (increasing the average read count to 4.65 million).

	First Round Results			Second Round Results		
Number of serotypes per sample	% correct	% correct (excluding 9 culture failures)	% correct (excluding 15 culture, primer failures)	% correct	% correct (excluding 9 culture failures)	% correct (excluding 15 culture, primer failures)
1	Major: 71<br>Minor: N/A<br>Overall: 71	Major: 83<br>Minor: N/A<br>Overall: 83	Major: 100<br>Minor: N/A<br>Overall: 100	Major: 71<br>Minor: N/A<br>Overall: 71	Major: 83<br>Minor: N/A<br>Overall: 83	Major: 100<br>Minor: N/A<br>Overall: 100
2	Major: 73<br>Minor: 49<br>Overall:61	Major: 84<br>Minor: 56<br>Overall: 70	Major: 96<br>Minor: 69<br>Overall: 80	Major: 76<br>Minor: 65<br>Overall: 70	Major: 88<br>Minor: 75<br>Overall: 81	Major: 100<br>Minor: 86<br>Overall: 93
3 or more	Major: 88<br>Minor: 49<br>Overall: 61	Major: 96<br>Minor: 55<br>Overall: 68	Major: 100<br>Minor: 57<br>Overall: 71	Major: 88<br>Minor: 67<br>Overall: 74	Major: 96<br>Minor: 76<br>Overall: 82	Major: 100<br>Minor: 78<br>Overall: 85
all	Major: 79<br>Minor: 49<br>Overall: 61	Major: 88<br>Minor: 55<br>Overall: 70	Major: 98<br>Minor: 59<br>Overall: 75	Major: 80<br>Minor: 66<br>Overall: 72	Major: 90<br>Minor: 76<br>Overall: 82	Major: 100<br>Minor: 81<br>Overall: 93

Table S2. PneuCarriage sensitivity results for all 80 samples.

Sequencing indices used for multiplexing

Samples from 96-well plates were barcoded using standard Illumina multiplexing PCR primers,

Index 1 Read: `5′ CAAGCAGAAGACGGCATACGAGAT[i7]GTCTCGTGGGCTCGG`

Index 2 Read: `5′ AATGATACGGCGACCACCGAGATCTACAC[i5]TCGTCGGCAGCGTC`

where the following i5 and i7 indices were used for the samples in each well:

i5 Indices			i7 Indices	
**Row**	**Index**		**Column**	**Index**
A	TAGATCGC		1	TCGCCTTA
B	CTCTCTAT		2	CTAGTACG
C	TATCCTCT		3	TTCTGCCT

D	AGAGTAGA		4	GCTCAGGA
E	GTAAGGAG		5	AGGAGTCC
F	ACTGCATA		6	CATGCCTA
G	AAGGAGTA		7	GTAGAGAG
H	CTAAGCCT		8	CCTCTCTG
			9	AGCGTAGC
			10	CAGCCTCG
			11	TGCCTCTT
			12	TCCTCTAC

Table S3. Illumina sequencing indices used for sample pooling.

References

1. Bentley SD, Aanensen DM, Mavroidi A, Saunders D, Rabbinowitsch E, Collins M, et al. Genetic analysis of the capsular biosynthetic locus from all 90 pneumococcal serotypes. PLoS Genet. 2006 Mar;2(3):e31. PubMed PMID: 16532061; PubMed Central PMCID: PMC1391919.

2. Kapatai G, Sheppard CL, Al-Shahib A, Litt DJ, Underwood AP, Harrison TG, et al. Whole genome sequencing of *Streptococcus pneumoniae*: development, evaluation and verification of targets for serogroup and serotype prediction using an automated pipeline. PeerJ. 2016 Sep 14;4:e2477. PubMed PMID: 27672516; PubMed Central PMCID: PMC5028725.
